# Fostering the exchange of real world data across different countries to answer primary care research questions: an UNLOCK study from the IPCRG

**DOI:** 10.1038/s41533-018-0075-9

**Published:** 2018-03-08

**Authors:** Liza Cragg, Siân Williams, Thys van der Molen, Mike Thomas, Jaime Correia de Sousa, Niels H. Chavannes

**Affiliations:** 1 International Primary Care Respiratory Group, Aberdeen, UK; 2 Department of General Practice, University Medical Center Groningen, University of Groningen, Groningen, The Netherlands; 3 Faculty of Medicine, Primary Care and Population Sciences, University of Southampton, Southampton, UK; 4 Life and Health Sciences Research Institute (ICVS), School of Medicine. ICVS/3B’s - PT Government Associate Laboratory, University of Minho, Braga, Portugal; 5 Department of Public Health and Primary Care, Leiden University Medical Centre, Leiden, The Netherlands

## Abstract

There is growing awareness amongst healthcare planners, providers and researchers of the need to make better use of routinely collected health data by translating it into actionable information that improves efficiency of healthcare and patient outcomes. There is also increased acceptance of the importance of real world research that recruits patients representative of primary care populations and evaluates interventions realistically delivered by primary care professionals. The UNLOCK Group is an international collaboration of primary care researchers and practitioners from 15 countries. It has coordinated and shared datasets of diagnostic and prognostic variables for COPD and asthma to answer research questions meaningful to professionals working in primary care over a 6-year period. Over this time the UNLOCK Group has undertaken several studies using data from unselected primary care populations from diverse contexts to evaluate the burden of disease, multiple morbidities, treatment and follow-up. However, practical and structural constraints have hampered the UNLOCK Group’s ability to translate research ideas into studies. This study explored the constraints, challenges and successes experienced by the UNLOCK Group and its participants’ learning as researchers and primary care practitioners collaborating to answer primary care research questions. The study identified lessons for future studies and collaborations that require data sharing across borders. It also explored specific challenges to fostering the exchange of primary care data in comparison to other datasets such as public health, prescribing or hospital data and mechanisms that may be used to overcome these.

## Primary care research: Using big data in chronic lung disease studies

Effective management of international primary care data sharing shows great potential to inform clinical practice for multiple diseases. The International Primary Care Research Group (IPCRG) was established in 2000 to progress sharing of big data between countries and support associated research projects. Liza Cragg of IPCRG in the UK and co-workers, reported on progress and limitations faced by one research group, UNLOCK, who are investigating the long-term impacts of chronic lung diseases. Cragg’s team interviewed UNLOCK members and analyzed results of individual research studies undertaken between 2010 and 2016. Constraints highlighted by researchers included inconsistencies across datasets, ethical concerns about data use, difficulties organizing international working groups and the voluntary nature of IPRCG work. Progress was slower than anticipated but several successful studies highlighted the potential for primary care data sharing to provide valuable insights.

## Introduction

The founding ambitions of the International Primary Care Respiratory Group (IPCRG) in 2000 included sharing data across countries to understand the real world similarities and differences in primary care management of chronic respiratory disease in different countries and to create large longitudinal datasets more rapidly.^1^ The UNLOCK Group (uncovering and noting long-term outcomes in chronic obstructive pulmonary disease (COPD) and asthma to enhance knowledge) is one of the projects IPCRG members established to achieve this ambition. It is an on-going international collaboration between primary care researchers and practitioners to coordinate and share datasets of relevant diagnostic and prognostic variables for COPD and asthma management, which began in 2010. It builds on the IPCRG’s published research needs, which called for research that evaluates interventions realistically delivered within primary care and draws conclusions meaningful to professionals working within primary care.^2,3^ The UNLOCK Group summary protocol was published in the *Primary Care Respiratory Journal* in 2010.^4^

This study explored the lessons from 6 years of the UNLOCK Group’s collaboration through: structured telephone interviews with key informants; an online questionnaire for participants using Survey Monkey; analysis of key documents the collaboration produced; and analysis of the datasets to which participants had access. The study methods are explained in more detail in the methods section which is situated after the discussion section.

The objectives of the study were:

1. To describe and classify the successes and motivation of participants in the UNLOCK Group in seeking to share real world data across regional and country borders to answer research questions and how these have been captured/built upon;
2. To describe and classify the constraints experienced by participants in the UNLOCK Group in seeking to share real world data across regional and country borders to answer research questions and how these have been overcome;
3. To identify methods to improve the effectiveness of future studies involving data sharing across countries to inform future research collaborations;
4. To describe other impacts of the UNLOCK Group, including the development of electronic recording systems, data validation and data extraction.

## Results

### Development of the UNLOCK Group and its participants

The first meeting of the UNLOCK Group took place in September 2010. At this meeting, the UNLOCK Group outlined a protocol summary which was then finalised and published.^5^ Key milestones in the UNLOCK Group’s development are described in Table 1. Early meetings were organised and financed by the IPCRG. The IPCRG Executive Officer also provided support for fundraising and for the development of a governance mechanism for UNLOCK. This governance mechanism provided the IPCRG Board with overall responsibility of the UNLOCK Group’s progress and performance, both financially and scientifically. Two consecutive unrestricted grants from Novartis enabled the UNLOCK Group to appoint a part-time project manager from January 2013 to May 2016 and part-time researcher from March 2014 to March 2016. From 2013 an IPCRG Steering Committee provided leadership and progress oversight. The UNLOCK Group continued to meet in person biannually.

**Table 1:** The UNLOCK Group chronology of key developments (2010–2015)

Year	UNLOCK meetings	Key developments	Publications
2010	June 2010– September 2010	Initial meeting agreed concept and confirmed support Extended meeting to agree how to work Potential research questions discussed	UNLOCK Abstract in *Primary Care Respiratory Journal*, 2010
2011	May 2011–September 2011	Three initial UNLOCK Group studies agreed and start Fundraising gets underway	
2012	April 2012	First Novartis grant of €60,000 Agreement of small grants to support each UNLOCK study Project to develop a common syntax for recoding agreed as Rosetta Agreement to extend the UNLOCK Group to include asthma	
2013	March 2013 May 2013 November 2013	Project manager appointed Niels Chavannes becomes the UNLOCK Group Chairperson UNLOCK Group only spaces on IPCRG web platform launched UNLOCK Steering Committee formed In depth discussion of block to progress and how to overcome these Agreement for UNLOCK Researcher Second Novartis grant of €140,000 UNLOCK studies on morning symptoms, comorbidities, asthma RCT validity and implementation of COPD treatment agreed	Lancet Letter “GOLD COPD categories are not fit for purpose in primary care”, March 2013
2014	May 2014–September 2014	UNLOCK Group Researcher starts UNLOCK Group study guidance prepared UNLOCK Group study on cost effectiveness of COPD and asthma treatment was endorsed UNLOCK Group study on implementation of COPD treatment stopped	UNLOCK study published in *PLOS ONE*
2015	May 2015–September 2014	UNLOCK Group Researcher extended for an additional year UNLOCK Group study on ACOS agreed	

Participation in the UNLOCK Group was initially conceived as being limited to members who could contribute primary care patient data on COPD that met inclusion criteria and extended to include primary care patient data on asthma in 2012. However, in practice individuals participated in meetings even if they did not have access to a database. Therefore, participation has been and continues to be based on interest in the work of the UNLOCK Group. Over the first 6 years approximately 26 individuals from 15 countries participated in the UNLOCK Group’s work. Table 2 lists these participants. Thirteen of the participants have a combined research/academic and clinical role, 10 have a research/academic role only and three have a clinical role only.

**Table 2:** Summary of the UNLOCK Group participants and their participation

	Name of individual	Research (R) or clinical (C) role or both (R &C)	Country	Number of UNLOCK Group meetings participated in	Number of UNLOCK Group studies to which data was contributed
1	Oleksii Korzh	R &C	Ukraine	0	1
2	Ana Moran	C	Spain	3	0
3	Andrew Cave	R	Canada	7	0
4	Antonius Schneider	R	Germany	1	0
5	Arnulf Langhammer	R &C	Norway	5	0
6	Bjorn Stallberg	R &C	Sweden	13	4
7	Bruce Kirenga	R &C	Uganda	1	0
8	Job van Boven	R	Spain/ Netherlands	2	2
9	Barbara Yawn	R	USA	1	0
10	David Price	R &C	UK/Singapore	8	4
11	Komalti Apte	R &C	India	2	0
12	Janwillem Kocks	R &C	Netherlands	10	6
13	Jaime Correia de Sousa	R &C	Portugal	8	1
14	Javiera Corbalan	C	Chile	2	0
15	Karin Lisspers	R &C	Sweden	10	4
16	Lynn Josephs	R	UK	5	0
17	Miguel Roman Rodriguez	C	Spain	8	2
18	Niels Chavannes	R &C	Netherlands	13	7
19	Peymane Adab	R	UK	2	1
20	Ioanna Tsiligianni	R &C	Greece	11	4
21	Rachel Jordan	R	UK	7	1
22	Rupert Jones	R &C	UK	8	1
23	Stefan Karrasch	R	Germany	1	0
24	Thys van der Molen	R &C	Netherlands	10	6
25	Mike Thomas	R	UK	8	1
26	Alan Crockett	R	Australia	1	0

### The data used by the UNLOCK Group

An analysis of datasets shows that by early 2016 participants in the UNLOCK Group had access to 14 datasets compared to 10 datasets available when the Group was established. Over this time one had become out-dated and five were added. These datasets included data from nine countries (Sweden, Spain, Ukraine, Canada, Greece, UK, Netherlands, Portugal, and India). They included data from 3.8 million primary care patients, of whom 800,000 were patients with asthma and 216,000 were patients with COPD. Appendix 1 lists the datasets and their key features. Several were large routine primary care datasets (HHR, OPCRD, CCPSN), others were cohort studies (Praxis, BLISS, rural Crete) and pragmatic clinical trials in primary care: Bocholtz study. Ten of the datasets were used in at least one UNLOCK study.

All datasets included some common variables such as: diagnosis, patient demographics; patient reported outcomes including health status and symptoms; spirometry; prescribed medication; exacerbations and smoking status. Some also included data on physician demographics, vaccinations, patient socioeconomic status and secondary care use.

The online questionnaire asked the UNLOCK Group participants about charges for using the data, ownership, governance and requirements for ethical approval for the datasets to which they had access for UNLOCK purposes. See Table 3 for a breakdown of results. For the majority of datasets there is no charge (57.1%) and where charges are applied this depends on the type of study (19%). Ethical approval requirements for using the datasets vary. The most common model (33.3%) is that ethical approval was secured at the beginning of the data collection and the data can be analysed and the anonymised results shared for new studies without seeking new approvals. The majority of the datasets are owned by the research institute (28.6%) or the practice that collects the data (23.8%). Governance arrangements for the datasets differ, with the most common model being governance by a committee (33.3%). Because of the variation of governance arrangements in place for these datasets, the UNLOCK participants decided it was not feasible to proceed with their original intention of pooling datasets. Instead they agreed to undertake data analysis separately using the same agreed analytical method for each study and then to pool findings.

**Table 3:** Arrangements for charging, ownership, governance, and ethical approval requirements for use of data used by the UNLOCK Group

	Number	Percentage of responses
*Is there a charge for suing the data for UNLOCK Group studies*		
Yes, there is a fixed charge for using the data	0	0.0
Yes, there is a charge for using the data that depends on the type of study	4	19.0
Sometimes there is a charge for using the data but this can be waived for UNLOCK Group studies	2	9.5
No charge for using the data	12	57.1
Other	3	14.3
*Who owns the data*		
The regional health authority	3	14.3
The national health authority	1	4.8
A research institute	6	28.6
The practice that collects it	5	23.8
A private company	1	4.8
Ownership arrangements are unclear	1	4.8
Other	4	19.0
*Who is responsible for the governance of the data*		
The regional health authority	4	19.0
The national health authority	0	0.0
A research institute	4	19.0
The practice that collects it	2	9.5
A committee	7	33.3
Governance arrangements are unclear	1	4.8
Other	3	14.3
*Requirements for ethical approval for use of the data*		
Ethical approval was secured at the beginning of the data collection and the data can be shared for new studies without seeking new approvals	3	14.3
Ethical approval was secured at the beginning of the data collection and the data can be analysed and the anonymised results shared for new studies without seeking new approvals	7	33.3
Ethical approval was secured at the beginning of the data collection and the data can be analysed and the anonymised results shared but new ethical approval is required for new study questions	3	14.3
New ethical approvals are required every time the data is used for a new study	3	14.3
Ethical arrangements are unclear	1	4.8
Other	4	19.0

### UNLOCK studies

By November 2016 eight studies initiated by the UNLOCK Group had result in publications. These are listed in Table 4. Ideas for additional studies were regularly discussed at UNLOCK meetings. A further seven study proposals were presented by participants in the UNLOCK Group, given provisional support, discussed at several UNLOCK meetings but were not ultimately pursued. One study idea was taken forward outside of the UNLOCK Group by one of the participants with other partners. This raised the challenge of intellectual property of ideas, reinforcing the need for building trust between the UNLOCK Group participants and for agreeing modus operandi. As a result a document specifying principles of engagement for participation in the UNLOCK Group was developed by the UNLOCK Steering Committee. These were distributed to all UNLOCK participants, although no formal signature was required.

**Table 4:** UNLOCK Group publications as of November 2016 Study

	Lead author	Co-authors	Publications/status
UNLOCK: uncovering and noting long-term outcomes in COPD to enhance knowledge	Niels H Chavannes	Bjorn Stallberg, Rupert C. M. Jones, Ioanna G. Tsiligianni, Karin Lisspers, Thys van der Molen, JanWillem H. Kocks, Miguel Roman, Ana Moran, Arnulf Langhammer, Alan Crockett, Andrew Cave, Sian Williams	UNLOCK Abstract published in *Primary Care Respiratory Journal*, 2010
Multi-component assessment of chronic obstructive pulmonary disease: an evaluation of the ADO and DOSE indices and the global obstructive lung disease categories in international primary care data sets	Rupert C Jones	David Price, Niels H Chavannes, Amanda J Lee, Michael E Hyland, Björn Ställberg, Karin Lisspers, Josefin Sundh, Thys van der Molen and Ioanna Tsiligianni	Article published in *npj Primary Care Respiratory Medicine*, April 2016; Abstract in *Primary Care Respiratory Journal*, April 2012; *Lancet Letter* “GOLD COPD categories are not fit for purpose in primary care”, March 2013
Primary care patients compared with large pharmaceutically-sponsored COPD studies: an UNLOCK validation study	Annemarije L. Kruis	Bjorn Stallberg, Rupert C. M. Jones, Ioanna G. Tsiligianni, Karin Lisspers, Thys van der Molen, JanWillem H. Kocks, Niels H. Chavannes	Article published in *PLOS ONE*, March 2014
Are pharmacological randomised controlled clinical trials relevant to real-life asthma populations? An UNLOCK study from the IPCRG	Karin Lisspers	Pedro Teixeira, Coert Blom, Janwillem Kocks, Björn Ställberg, David Price and Niels Chavannes	Protocol published in *npj Primary Care Respiratory Medicine*, April 2016
Predictors of cost-effectiveness of selected COPD treatments in primary care: an UNLOCK study from the IPCRG	Job FM van Boven,	Miguel Román-Rodríguez, Janwillem WH Kocks, Joan B Soriano, Maarten J Postma and Thys van der Molen	Protocol published in *npj Primary Care Respiratory Medicine*, April 2016
The prevalence of night and morning symptoms in primary care COPD patients: how do they relate to COPD health status and disease severity?	Ioanna G. Tsiligianni	Esther, Metting Thys van der Molen, JanWillem H. Kocks, Niels H. Chavannes	Published in *npj Primary Care Respiratory Medicine*, July 2016
The prevalence of comorbidities in COPD patients and their impact on the quality of life and COPD symptoms in primary care patients	Björn Ställberg	Pedro Teixeira, Coert Blom, JanWillem Kocks, Karin Lisspers, David Price and Niels Chavannes, Job van Boven, Miguel Roman-Rodriguez, Oleksii Korzh, Rachel Jordan	Protocol published in *npj Primary Care Respiratory Medicine*, November 2016
Fostering the exchange of real life data across different countries to answer primary care research questions: an UNLOCK study from the IPCRG	Liza Cragg	Siân Williams, Thys van der Molen, Mike Thomas, Jaime Correia de Sousa, Niels H. Chavannes	Protocol published in *npj Primary Care Respiratory Medicine*, September 2016

### Participants’ perceptions of the UNLOCK Group

Interviews were held with 12 key informants to identify their initial motivations to become involved with the UNLOCK Group and to determine what they wanted to achieve through their involvement. The most frequently cited motivation was to improve understanding of the primary care perspective and real world research, which six informants mentioned. The opportunity to learn from other countries (*n* = 5); the social aspect of collaboration (*n* = 5); the potential to improve patient outcomes (*n* = 3); and the ability to add power to existing data (*n* = 2) were also relevant factors.

Informants were asked if they thought the UNLOCK Group was achieving its primary purpose, to provide evidence to underpin the primary care approach to diagnosis, assessment and broad management strategies. All said the UNLOCK Group had worked within this purpose, although seven said they thought it was a long term and ambitious goal.

Seventy-nine percent of respondents (15/19) of the online questionnaire perceived the UNLOCK Group to be “very effective” or “effective” in its primary purpose, while the remaining 21 percent (4/19) said they thought it had “not been very effective.” Respondents were also asked to rate what they considered to be the main successes of the UNLOCK Group from a list of pre-specified responses. As shown in Table 5, the generation of ideas for progression outside UNLOCK, and bringing together colleagues from different countries, were rated most highly as the main successes of the Group, followed by the UNLOCK studies.

**Table 5:** UNLOCK main successes

Answer options: main successes	Rating Average
Generating new ideas for research questions that are taken forward outside the UNLOCK Group	4.05
Bringing together colleagues from different countries	3.95
The UNLOCK Group studies that have been completed	3.84
Creating a forum to share data issues with colleagues informally	3.79
Encouraging the development of new datasets	3.68
Others	1.68

All key informants were also asked an open question about what they consider to be the main successes of the UNLOCK Group. The themes raised by more than one informant in order of their popularity were:

- The UNLOCK Group studies that have been completed (10/12).
- An inclusive and sustained network (9/12).
- Raising the profile and credibility of real world research (7/12).
- Successfully working across countries (6/12).
- The ideas generated (3/12).

Key informants were also asked to describe the impact of the UNLOCK Group’s work. The themes raised by more than one informant in order of their popularity were:

- The development of a network for primary care research across countries (9/12).
- Building for future work (4/12).
- Proving international research in primary care could be done (3/12).
- Not possible to say without reviewing the studies in detail (2/12).
- The impact of UNLOCK Group had been limited (2/12).

The majority of key informants interviewed stated that progress had been slower than expected. When asked about the main problems, the majority (7/12) indicated that the UNLOCK Group wasn’t a priority for the participants because of the demands of their main clinical and/or research role). Half of the informants also said data differences had hampered research output. Other issues raised were: the extent to which research questions were being driven by the availability of data; whether the UNLOCK Group’s research questions could best be answered by combining different, heterogeneous datasets from different countries as opposed to a large dataset from a single source; an over-reliance on data from three countries; inadequate resources to progress studies; a tendency amongst participants to generate ideas rather than carry studies through to completion; not enough time in meetings; difficulties coordinating study contributors; ethical and cultural barriers; language differences; lack of leadership from the UNLOCK Steering Committee; delineating what is UNLOCK Group when participants have multiple roles; and having too many small datasets.

The online questionnaire asked UNLOCK Group participants to agree or disagree with a range of statements about UNLOCK Group meetings. All respondents either agreed or agreed strongly with the statement “I find UNLOCK Group meetings really useful because I get to hear about the research my colleagues are doing.” There was also majority agreement with the statement “I find UNLOCK Group meetings really useful because I learn about the clinical consultation, essential data collection and coding in other countries which helps me understand similarities and differences better.” No respondents agreed with the statement “I do not attend meetings as I do not find UNLOCK Group meetings useful.” Table 6 shows the statements and average response, with higher scores indicating stronger levels of agreement.

**Table 6:** Perceptions of UNLOCK Group meetings

Answer options: UNLOCK Group meetings	Disagree	Neither agree nor disagree	Agree	Agree strongly
I find UNLOCK Group meetings really useful because I get to hear about the research my colleagues are doing	0 (0%)	0 (0%)	8 (42%)	11 (58%)
I find UNLOCK Group meetings really useful because I learn about the clinical consultation, essential data collection and coding in other countries which helps me understand similarities and differences better	1 (5%)	3 (16%)	7 (37%)	8 (42%)
I find UNLOCK Group meetings really useful because I reflect on how to improve dataset management	4 (21%)	4 (21%)	6 (32%)	5 (26%)
I find UNLOCK Group meetings really useful because I get the opportunity to think about the advantages and disadvantages of different coding approaches	5 (26%)	5 (26%)	5 (26%)	4 (22%)
I have used the learning at UNLOCK Group meetings to influence the design of electronic health records in my practice/region/country	7 (37%)	5 (26%)	4 (21%)	3 (16%)
I would like to attend UNLOCK Group meetings but I am unable to prioritise attending them because of other work commitments.	11 (58%)	2 (10.5%)	4 (21%)	2 (10.5%)
I do not attend meetings as I do not find UNLOCK Group meetings useful.	16 (84%)	3 (16%)	0 (0%)	0 (0%)

### Action to overcome challenges

Analysis of the minutes of UNLOCK Group meetings showed some studies were discussed regularly at UNLOCK Group meetings over four years. Meeting minutes also highlighted that participants discussed how to progress studies more effectively on several occasions. Over the years 2010–2016, participants in the UNLOCK Group introduced new ways of working to overcome barriers to progress. These included:

- Arranging meetings to coincide with existing conferences.
- Using PhD students to progress studies.
- Paying small grants to lead authors to facilitate studies.
- Setting up a private discussion space on the IPCRG web platform.
- Dedicated project management.
- Setting up a Steering Committee.
- Testing the feasibility of a single data exchange portal.
- Funding a part-time researcher managed by a Steering Committee member.
- Defining processes and templates for developing and implementing an UNLOCK Group study.

Interviews with key informants revealed adoption of a pragmatic approach to solving problems as they arose. When asked if this approach to had been effective, the broad consensus was “partly”. Reasons suggested why these had not been more effective included:

- The researchers collaborating on UNLOCK Group studies are not located in the same place.
- There are lots of different expertise needed to progress the UNLOCK Group studies including methodology, epidemiology, statistics, project management, fundraising, writing and it is not possible to get all of these from one person but the UNLOCK Group does not have the resources to fund a team.
- The UNLOCK Group meetings are big and fast which can be a barrier to detailed discussions on study findings.
- While new processes have led to the improvement of the articulation of study ideas into written protocols, these still need to be improved.

### Future development of the UNLOCK Group

Interviews with key informants sought to clarify what lessons of the UNLOCK Group’s work could be applied to future studies. Three primary themes raised by more than one informant in order of their popularity were:

- Do not try to pool data as it is not feasible (5/12).
- Do not use existing data as a starting point (4/12).
- Improve organisation and professionalism (3/12).

Respondents to the online questionnaire (*n* = 19) were asked what they thought were the priorities for future UNLOCK Group studies. The topics prioritised by most respondents were COPD management in primary care (prioritised by 95% of respondents, comorbidities (74%), asthma management in primary care (68%) and COPD diagnosis in primary care (53%). Respondents were also asked to indicate whether they agreed with a number of pre-specified options for the development of the UNLOCK Group. The three options which scored the most support were “it needs new countries to join to bring additional data from more diverse contexts” (supported by 53% or respondents), “UNLOCK Group participants need to make better use of their existing data by doing more studies” (53%) and “the UNLOCK Group participants need to focus more on supporting each other and new participants to create and improve datasets” (47%).

## Discussion

### Main findings

#### Challenges and constraints

This study revealed three primary themes that influenced the challenges and constraints experienced by the UNLOCK Group between 2010–2016. The first was data constraints which impacted on the comparability of data. These constraints included: variable dataset size and accuracy with small, detailed datasets from cohort studies being more accurate than large routine datasets; datasets including different variables or the same variables using different codes and definitions; and the availability of incentives in some countries for diagnosis and treatment. These data comparability challenges raise concerns about the internal validity of studies. As a result, discussion of studies at the UNLOCK Group meetings increasingly stressed the importance of the UNLOCK Group studies comparing different countries’ experiences, rather than combining data to achieve more power. There was also a perception amongst participants of constraints due to governance and ethical requirements for using some datasets. In practice it is not clear that these constraints prevented the use of data, although they sometimes caused delays. Not having required data variables was more of a constraint for participants contributing to studies. In addition, contributing data to a study required time from participants.

The second theme was challenges that arose from participants from several countries working as a group including: different expectations of how meetings should be used; difficulties in using English as the working language for some non-native English speakers; using different processes for developing study proposals; and cultural differences in how to undertake methodological scrutiny without appearing overly critical.

The third theme of constraints related to participants’ lack of time. The UNLOCK Group participants have many demands on their time due to their main clinical and/or research roles, and participation in the UNLOCK Group is entirely voluntary. The voluntary and collaborative nature of the UNLOCK Group meant studies progress as fast as the slowest responder. If there were problems with studies they were brought back to the next meeting for further discussion. This led to some frustration that some studies were not progressing fast enough, with a sense that some studies were repeatedly discussed at the UNLOCK Group meetings.

### Strategies to overcome challenges and constraints

The UNLOCK Group participants adopted various strategies to overcome challenges using a pragmatic approach to solving problems as they arose. These strategies were partly effective but had their own limitations. In addition to data cleaning and validation, templates for developing and implementing an UNLOCK Group study were developed to ensure quality and consistency. While this enabled improvement in proposals for the later studies, protocols for some of earlier studies were not sufficiently well defined. This led to one study being abandoned after significant time and work. To overcome time constraints the UNLOCK Group participants used PhD students and more junior researchers to progress studies. Although this greatly facilitated the progress of the UNLOCK Group studies, it was only an option for the UNLOCK Group participants affiliated to academic institutions with PhD students engaged in primary care respiratory research, which remains a small academic discipline. The UNLOCK Group participants also agreed to make available small grants to lead authors to facilitate studies. These enabled lead authors to lever in additional resources from their own institutions where available, but were not adequate to cover the costs of their own time. A Steering Committee was convened to take decisions outside meetings but this did not have a role in progressing studies. Later a part-time researcher managed by a Steering Committee member was recruited. However, this did not speed up progress as much as anticipated, perhaps because the researcher was not co-located with the first authors. A part-time project manager was engaged which improved communication and co-ordination between participants. To facilitate progress outside meetings a private discussion space on the IPCRG web platform was set up. However, most participants preferred face-to-face interaction.

### Box 1: Case studies exploring factors for successful UNLOCK studies

The UNLOCK Group study “Primary care COPD patients compared with large pharmaceutically-sponsored COPD studies: an UNLOCK validation study” was initiated in September 2011. Final results were presented in May 2013 and it was published in *PLOS ONE* in March 2014.^6^ Despite the fact that it included data from seven UNLOCK datasets, it was completed and published promptly. Critical factors for which enable this study to be successful concluded are:

- The central hypothesis of the study was clearly articulated at the start;
- The study was of identifiable clinical relevance which motivated the study team;
- The UNLOCK Group participant leading the study identified a dedicated research team in their own institution to undertake the analysis. This included a goal-oriented PhD student whom they directly supervised;
- With the support of the UNLOCK Group participant, this research team were able to mobilise their counterparts in partner institutions to ensure data was provided promptly, rather than relying exclusively on the UNLOCK Group participants themselves.

In contrast, the UNLOCK Group study “Comparing GOLD stages between countries” was initiated in September 2011. Interim analysis of four datasets was undertaken and results presented in March 2013. Interim findings were discussed regularly at UNLOCK Group meetings through 2014 and 2015 with suggestions made for the improvement of the findings. The study was finally abandoned in September 2016 when considered no longer viable. Reasons identified include:

- There were problems with internal validity due to combining large and small datasets and with differences in the definitions of variables between datasets;
- The lead UNLOCK Group participant did not have a research team on site to support the analysis;
- The research question did not motivate enough researchers to participate;
- Because the study took so long, its clinical relevance was reduced by changes to management guidelines.

### Main successes and impacts

In the six year period that this study covers, the UNLOCK Group undertook nine studies on the diagnosis and management of COPD, asthma and ACOS in primary care. These had resulted in eight published journal articles (with more in press) and many presentations at leading international conferences by mid-2016. In addition to these outputs, the UNLOCK Group developed a network of primary care researchers across 15 very different countries which was sustained for over six years. This brought access to data from 3.8 million primary care patients across 10 countries, creating potential for multi-country studies involving large numbers of patients.

The UNLOCK Group participants broadly agreed that the impact of the studies on practice was low. However, translating research into clinical practice is widely recognised as being extremely complex, difficult and time consuming. Studies have found that it takes around 17 years to translate research evidence into changes in care that benefited patients.,^7–9^ Therefore, it would be overly ambitious to expect the UNLOCK Group to have demonstrable impact on clinical practice.

In addition, the participants described other significant impacts that the UNLOCK Group has achieved. These included raising the profile and credibility of real world research and proving it is possible for an international collaboration to do primary care research. The UNLOCK Group acted as a catalyst for the development of new national primary care datasets that would be fit for routine and research use. The UNLOCK Group datasets are now being used to validate the first European COPD atlas. In addition, it stimulated ideas and collaborations that have been taken forward outside the UNLOCK Group. These include collaborations on significant externally funded research projects; the UNLOCK Group participants acting as co-authors to other participants on studies; the UNLOCK Group participants providing external supervision for PhD students from each other’s institutions; and collaborations at research conferences and symposia.

The slow progress with some of the UNLOCK Group’s studies and the frustration that more had not be achieved is partly due to the limitations inherent in the collaborative model, which relies on voluntary participants. This means it is difficult to enforce deadlines. The UNLOCK Group also relied on the skills and capacities participants brought to lead studies, supplemented by a part-time project manager and researcher. It became clear that more specialist and dedicated expertise in epidemiology, methodology, statistical analysis and medical writing was needed but the UNLOCK Group did not have sufficient resources to fund a team with these skills.

There was consensus among participants that some of the frustration resulted from unrealistically high expectations about what could be achieved in the beginning and that they were learning by doing so identifying the challenges and constraints had been part of the development process.

### Specific challenges for primary care data

The UNLOCK Group participants interviewed said that many of the challenges the Group experienced were common to other medical and healthcare research, for example, consent, ethics, data ownership. However, they also suggested there were some specific challenges to fostering the exchange of primary care data in comparison to other datasets, such as secondary care. These included the large number of conditions that are diagnosed and managed in primary care which require very many different codes and different primary care coding systems. In addition, some participants suggested routine data collected in primary care may be of more variable quality than that collected in secondary care, with the lack of trained coders, the range of codes and payment incentives for quality indicators impacting on data accuracy. There were also particular ethical and governance issues involved in using routine data because it has not been collected for research purposes. Some informants said that variations in the way primary care is structured and incentivised between countries are greater than other areas of speciality and that there is less capacity and expertise for research in primary care than in other areas.

### Interpretation of findings in relation to previously published work

Recent work has highlighted that large scale collection and analysis of routine data, including patients’ experiences and outcomes, has become common place in health care systems although these large datasets are not used to their full potential.^10^ Research has shown that multiple large databases can be used effectively to increase power, to assess outcomes, or to study diverse populations and has proposed conceptual models to facilitate the process of analyzing data from multiple databases.^11^ Research has also demonstrated that real world data has an important role to play in the evaluation of burden of disease, treatment patterns, compliance, persistence, and health outcomes of different treatments.^12^

This study adds to this work by demonstrating the value of researchers and clinicians collaborating to share real world data across regional and country borders to answer research questions studies and by exploring the factors that enable such collaboration in practice. It also identifies the knowledge developed by the UNLOCK Group to improve the effectiveness of future collaborations and studies.

### Strengths and limitations of this study

Limitations to this study and measures taken to mitigate these include:

- The interviews and analysis were carried out by the UNLOCK Group project manager, who was funded by the project and therefore had a potential conflict of interest in demonstrating positive outcomes. This was mitigated by peer review of all questionnaires and presentation of the draft findings at an UNLOCK Group meeting. In addition, an abstract on the interim findings was peer-reviewed and presented at the IPCRG 8th World Conference, where independent judges voted it one of the top three abstracts out of 137.
- Some participants in early UNLOCK Group meetings who stopped being actively involved did not complete the online questionnaire therefore the motivations and challenges of those who chose to leave is not fully documented.
- The findings show participants in the interviews and questionnaires had a very positive experience of the UNLOCK Group meetings as a safe place to explore ideas and to network with colleagues with similar mind-sets. There is a risk this reflects “groupthink” in these meetings, whereby participants try to minimise conflict and reach a consensus.^13^ However, this study also included analysis of minutes of meetings which demonstrate robust discussion and challenges to ideas and data presented.
- Individuals who have received documents related to the UNLOCK Group but who have not actually participated were not invited to complete the online questionnaire. Therefore, the study does not attempt to explore why some invited researchers did not participate.

### Implications for future research, policy, and practice

Better use and integration of routinely collected health data to develop population medicine approaches, risk stratification and segmentation to improve outcomes is essential. For example, high need high cost patients who experience a variety of complex health and functioning problems are among the 5% of patients who account for 50% of health care spending in the USA. Primary care data are essential to understanding how to better meet their health needs.^14^ In addition, primary care data capture multi-morbidities and consequently can help answer important questions on the diagnosis and management of patients with multi-morbidities. For example, a recent clinical audit in the UK found 80% of patients with a COPD diagnosis had a comorbidity.^15^ It can also help understand how best to address the determinants of health which are currently addressed inadequately by many healthcare providers.^16^

Research funders have recently prioritized the challenge of finding ways to break down data silos and translate data into actionable information.^17^ There is consensus in policy documents and research agendas that primary care has a key role in the fight against NCDs.^18–20^ Consequently, guidelines and pathways need to reflect primary care experience, priorities and needs, including the generation and incorporation of evidence about primary care approaches to diagnosis, assessment and effective management strategies.^21^ In addition, policy makers and funders have identified the need to improve the quality and availability of primary care data.^22^ Recent European health policy has also called for better use of existing data to formulate policies to improve the health of individuals and populations.^23,24^

At the same time, there is increased emphasis on the need for data to be shared between researchers to accelerate analysis and learning. However, challenges in building the culture and resources needed to support data sharing are considerable and include concerns that researchers in resource-poor settings will lose out to better-resourced researchers, fears that increased data sharing will create unacceptable risks for patients and the costs in terms of money and time of data sharing.^25^

The experience of the UNLOCK Group has shown that individual relationships and trust are strong motivator for individuals to collaborate. However, personal relationships and individual motivation alone are unlikely to be sufficient to deliver high quality research over a sustained period without significant dedicated resources. Individual professionals with experience of real world practice and access to data often lack the time and expertise to undertake research themselves. However, research institutions that have the resources and expertise do not have the same experience of real world practice. This means collaborations between those with access to the data and experience of practice and researchers need to be better supported financially to enable capacity building and knowledge exchange.

The UNLOCK Group has also shown there are complex issues in sharing data from primary care across countries including dataset size, representativeness, coding accuracy, significance of variables. There is “no one size fits all” solution for these. There are also ethical and governance issues which can make accessing routine data time-consuming and difficult. This means the research question needs to be right, requiring international comparison rather than pure power and a strong interest from collaborators. Data sharing is more complex that a single dataset study so methodological and statistical expertise is required.

Primary care data offers unique opportunities to explore symptoms, diagnosis and patient reported outcomes. Rather than merely supplementing typical approaches of disease specific guidelines, pathways and evidence generation, it can contribute to the development of newer concepts of functioning, health and disability with better capture patient perspectives and multi-morbidities. This reflects developments in family medicine teaching and can support shared decision-making. However, there are some specific methodological challenges in using this data. More work is needed to support the standardisation or datasets, including dictionaries of codes and definitions. Finally, the coordination and time that international collaborations such as the UNLOCK Group require should not be underestimated.

## Conclusions

From 2010 to 2016 the UNLOCK Group demonstrated a strong interest and willingness to engage in international research collaborations amongst primary care researchers and clinicians. This reflected a belief that shared learning between countries is important and primary care research has a unique value, including capturing comorbidities, patient reported outcomes and using a person-centred approach. The collaboration completed several important studies and achieved a wider impact by demonstrating that real world research across middle and high income countries is feasible. It also generated indirect benefits by instigating new research collaborations between institutions and individuals and new datasets. The complexity of sharing data across countries and coordinating the UNLOCK Group’s work meant that progress was slower than participants anticipated. However, most participants remained committed and confident that with the right support and financial and human resources it was possible to provide valuable insights and generate learning between countries on the diagnosis, treatment and prevention of respiratory diseases in the community which could be used to inform the production of better guidance and pathways for practising clinicians.

## Methods

### The study used a mixed methods design including the following

- A review of documents related to the UNLOCK Group was undertaken to develop the chronology of key events; to identify research ideas and how these progressed into studies; to identify challenges identified by the Group and action proposed to overcome these; and to identify who participated in the Group over the 6 years covered. The review identified documents on the IPCRG web platform related to the UNLOCK Group meetings and published articles produced by the UNLOCK Group. A list of the documents identified was then checked with the minutes of UNLOCK Group meetings to ensure all documents that had been used at those meetings were included. In addition, the UNLOCK Group Chairperson and the IPCRG executive director checked the list of documents to ensure none had been omitted. The review of documents was carried out by the UNLOCK Group project manager.
- A structured online questionnaire for UNLOCK Group participants was undertaken using Survey Monkey in January and February 2016. The questionnaire explored: the roles of participants; the data to which they have access; perceptions of the strengths of the UNLOCK Group; obstacles that limit participation in UNLOCK studies; how these can be overcome; and ideas about future priorities. The questionnaire was designed by the UNLOCK Group project manager and the IPCRG executive director. It was tested by two UNLOCK members and then refined based on their feedback. Twenty-seven participants in the UNLOCK Group were invited to complete the questionnaire. Of these 21 responded; a response rate of 77%. However, not all questions were completed by all respondents. Analysis of the responses to the questionnaire were based on the response rate for each question. Responses to the online questionnaire were analysed by comparing the percentage of respondents who gave each answer or agreed and disagreed with suggested statements and by rating participants’ prioritisation of suggested themes. Analysis was undertaken by the UNLOCK Group project manager and checked by the IPCRG executive director.
- Structured interviews with 12 UNLOCK Group participants, including Steering Committee members and lead authors for the UNLOCK Group studies, to explore the barriers to undertaking and completing these studies and how they can be overcome and the successes, impacts and the learning to date. The informants’ responses to each question were coded and thematically analysed to identify the key issues from the perspective of the participants. The frequency with which each issue was raised by informants was also analysed to explore to what extent perspectives were shared. Analysis was undertaken by the UNLOCK Group project manager and checked by the IPCRG executive director.
- A review of the datasets held by current participants in the UNLOCK Group, how these have changed over time and how many of these datasets have been used for the UNLOCK Group studies. Each participant in the UNLOCK Group was requested to provide information on defined characteristics of their dataset in September 2015. The number of patients included in these datasets and the variables they collected were compared with those available to the UNLOCK Group in 2012. Analysis was undertaken by the UNLOCK Group project manager and checked by the IPCRG executive director.

More details of methods can be found in the published study protocol.^26^

## Electronic supplementary material

Supplementary Information
