# Supplementary material for: Fostering the exchange of real world data across different countries to answer primary care research questions: an UNLOCK study from the IPCRG
Source: NPJ Prim Care Respir Med. 2018 Mar 8;28:8. doi: 10.1038/s41533-018-0075-9 (PMC5843627; doi:10.1038/s41533-018-0075-9)
Supplement: Supplementary file 1 — Supplementary Information [file 41533_2018_75_MOESM1_ESM.docx]

## Supplementary information

## Appendix 1: UNLOCK datasets

| **Country** | **Region** | **Name of participants in the UNLOCK Group** | **Name of dataset** | **Number of patients** | **Patients with asthma diagnosis** | **Patients with COPD diagnosis** | **Patients with asthma & COPD diagnosis** | **Data collection (period)** |
| --- | --- | --- | --- | --- | --- | --- | --- | --- |
| UK | Hampshire | Mike Thomas & Lynn Josephs | Hampshire Healthcare Record | 1.2 million | Not known yet | 15,000 | Not known yet | Jan 2011-Dec 2013 |
| UK | Birmingham | Rachel Jordan | BLISS | 1,500 | Yes | Yes | Yes | 2012-2016 |
| UK | UK wide | David Price | OPCRD | 2.3 million | 736,965 | 142,269 | 45,529 | 1980-2015 |
| Ukraine | Kharkov, Sumy, Poltava, Chernigov, Cherkassy, Rivne regions | Oleksii Korzh | GP data | 5,330 | 2,350 | 2,980 | No | 2012-2015 |
| Greece | Crete | Ioanna Tsiligianni | GP practice in rural Crete | 130 | 30 | 100 | No | 2012 - 2015 |
| Spain | Balearics | Miguel Roman | MAJORICA | 68,578 | 45,800 | 27,871 | 5,093 | 2011-2014 |
| Canada | All 10 Canadian provinces | Andrew Cave | CPCSSN | 250,000 | No | 10,043 | No | From 2008 |
| Portugal | Braga | Jaime Correia de Sousa | UNLOCK pilot | 205 | No | 205 | No | 2015 |
| The Netherlands | Groningen and parts of Friesland and Drenthe | Thys van der Molen & Janwillem Kocks | Asthma/COPD-service Groningen | 17,000 | Yes | Yes | Yes | From 2007 |
| The Netherlands | Southern Netherlands | Niels Chavannes | Bocholtz study | 154 | No | 154 | No | 2008 |
| The Netherlands | Leiden/  Hague | Niels Chavannes | General practice routine data | 9,265 | 2,100 | 7,000 | 165 |  |
| Sweden | Mid Sweden | Karin Lisspers and Bjorn Stallberg | Praxis cohort | 900 | 525 | 425 | Only if the patients above have both diagnosis | 2014 -2012 |
| Sweden | Mid Sweden | Karin Lisspers and Bjorn Stallberg | New cohort | 1,800 | 900 | 900 | Only if the patients above have both diagnosis | 2014 |
| India | Across India | Komalkirti Apte | CRF network | 2,500 | 1,135 | 534 | 8 | From 2011 |
